# Supplementary material for: Causal Relationship between Aging and Anorexia Nervosa: A White-Matter-Microstructure-Mediated Mendelian Randomization Analysis
Source: Biomedicines. 2024 Aug 16;12(8):1874. doi: 10.3390/biomedicines12081874 (PMC11351342; doi:10.3390/biomedicines12081874)
Supplement: Supplementary file 1 [file biomedicines-12-01874-s001.zip › Supplementary Figure/Figure S4/Singe SNP plot of MR analysis for TL to ubm-b-1463.pdf]

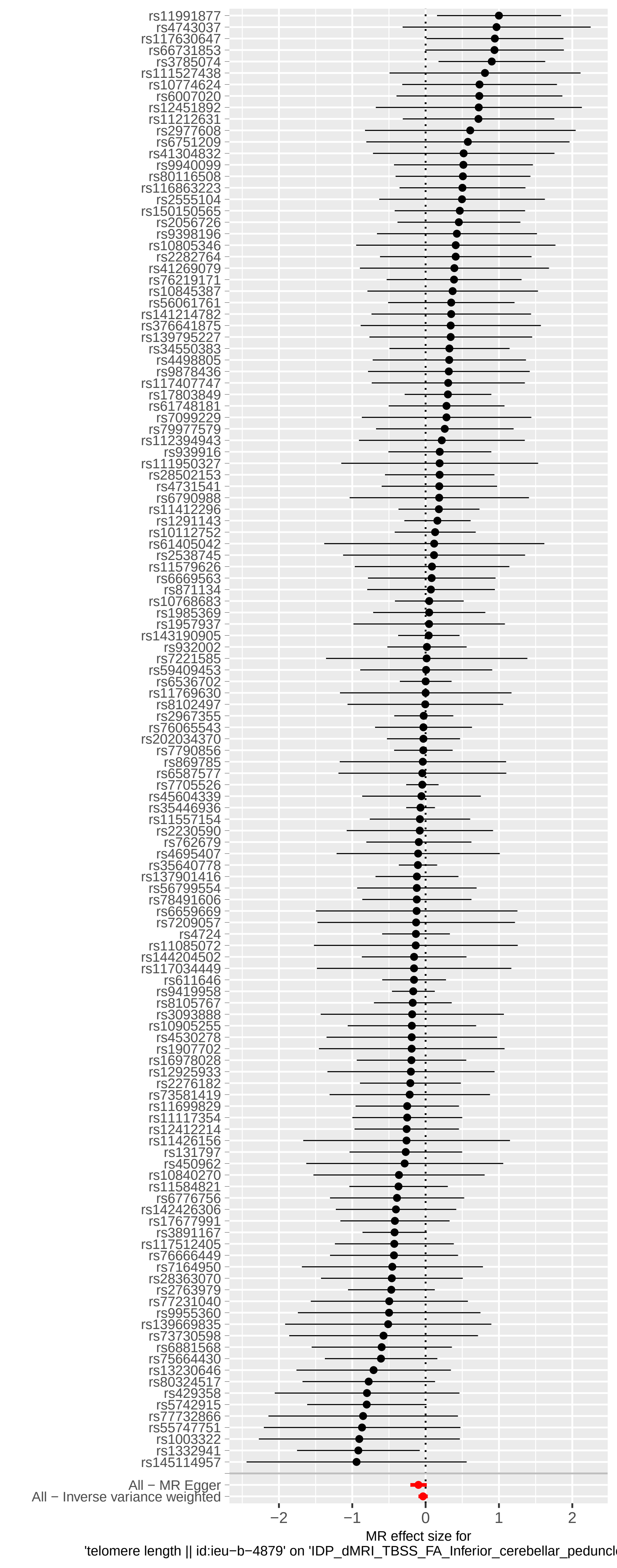

rs11991877
rs4743037
rs117630647
rs66731853
rs3785074
rs111527438
rs10774624
rs6007020
rs12451892
rs11212631
rs2977608
rs6751209
rs41304832
rs9940099
rs80116508
rs116863223
rs2555104
rs150150565
rs2056726
rs9398196
rs10805346
rs2282764
rs41269079
rs76219171
rs10845387
rs56061761
rs141214782
rs376641875
rs139795227
rs34550383
rs4498805
rs9878436
rs117407747
rs17803849
rs61748181
rs7099229
rs79977579
rs112394943
rs939916
rs111950327
rs28502153
rs4731541
rs6790988
rs11412296
rs1291143
rs10112752
rs61405042
rs2538745
rs11579626
rs6669563
rs871134
rs10768683
rs1985369
rs1957937
rs143190905
rs932002
rs7221585
rs59409453
rs6536702
rs11769630
rs8102497
rs2967355
rs76065543
rs202034370
rs7790856
rs869785
rs6587577
rs7705526
rs45604339
rs35446936
rs11557154
rs2230590
rs762679
rs4695407
rs35640778
rs137901416
rs56799554
rs78491606
rs6659669
rs7209057
rs4724
rs11085072
rs144204502
rs117034449
rs611646
rs9419958
rs8105767
rs3093888
rs10905255
rs4530278
rs1907702
rs16978028
rs12925933
rs2276182
rs73581419
rs11699829
rs11117354
rs12412214
rs11426156
rs131797
rs450962
rs10840270
rs11584821
rs6776756
rs142426306
rs17677991
rs3891167
rs117512405
rs76666449
rs7164950
rs28363070
rs2763979
rs77231040
rs9955360
rs139669835
rs73730598
rs6881568
rs75664430
rs13230646
rs80324517
rs429358
rs5742915
rs77732866
rs55747751
rs1003322
rs1332941
rs145114957
All − MR Egger
All − Inverse variance weighted
−2
−1
0
1
2
MR effect size for
'telomere length || id:ieu−b−4879' on 'IDP_dMRI_TBSS_FA_Inferior_cerebellar_peduncl
